# The Drain on the Hospitals' Special Departments

**Published:** 1914-08-29

**Authors:** 


### THE POSITION OF VOLUNTARY HOSPITALS.

### The Drain on the Hospitals' Special Departments.

Whilst it is perfectly true that a great deal of the routine medical and surgical work of the hospitals, particularly in regard to out-patients, could be carried on by volunteers from without—that is to say, by practitioners whose keenness leads them to come to the rescue of their medical schools—it is evident that some difficulty must arise in the administration of the special departments owing to the departure of many experts for military service. The position of the specialists in operative surgery, and the difficulty that must arise in dealing with the usual number of operations each week at the leading hospitals, was discussed in the last two issues of The Hospital, when it was pointed out that at one institution, for example, the entire surgical staff was attached to the Territorial Army, whilst at another only one junior member of the staff was left to carry on the entire operative work. Doubtless by reducing the operating-theatre lists to urgent cases only some satisfactory attempt to deal with the work for the present may be made. It is, however, to the needs of the other special departments of hospital work that it is now desired to draw attention. The training of specialists in ophthalmology, dental surgery, vaccine therapy, or radiography, for example, requires years of careful study and application, and it is quite out of the question that the places of experts in these and other branches who have had to leave their posts can be filled from the ranks of volunteer practitioners. That numerous specially trained physicians and surgeons have already left for military duty, and that others are about to go, is evident enough. Thus Westminster Hospital has already lost the director of its inoculation department, Dr. Carmalt-Jones, who has left to serve with the "Artists," as well as an eminent neurologist attached to its staff, Dr. Purves Stewart, who has joined the No. 4 General Territorial Hospital, based on King's College Hospital. Dr. Purves Stewart's services will also be necessarily missed from the West End Hospital for Nervous Diseases, in Welbeck Street, where for many years he has been a prominent worker and teacher. Further, Mr. Theodore Burns, senior anæsthetist to the Middlesex Hospital, is attached to the 3rd London General Hospital, whilst Dr. W. D'Este Emery, the bacteriologist, has mobilised with the 4th London General Hospital.

### Radiography.

One department of hospital work in particular which is bound to suffer very much in this connection is that for $x$-ray treatment and diagnosis, and already a number of $x$-ray experts have left for the front. In no previous war have radiographers had such an opportunity of assisting in the relief of the wounded as in the present campaign, for at no previous time has radiography been developed to such a pitch of excellence. At the time of the Boer War the $x$-ray apparatus was in its infancy, and although it was better developed to meet the requirements of military surgery in the Russo-Japanese campaign, the science of $x$-ray diagnosis had not then reached its present stage of technical perfection. It is no exaggeration to say that the $x$-ray specialists now taking the field will be the means of saving thousands of combatants from the horrors of permanent crippling and deformity, as well as of enabling surgeons to extract missiles in numberless instances where otherwise they would have to be left, with fatal results.

Amongst surgeons in charge of throat, ear, and nose departments who have already mobilised may be mentioned Mr. Somerville Hastings, F.R.C.S., of the "Middlesex," just promoted to a captaincy in the 3rd London General Hospital; and Mr. G. Nixon Biggs, of the Seamen's Hospital, Greenwich, and the Royal Waterloo Hospital. It is understood that Mr. Biggs is not organising any particular section in connection with his speciality, but at present is fulfilling the routine duties of an R.A.M.C. officer of the Territorial Army, in which he holds the rank of major.

### Dental Surgery.

It appears probable that the services of dental surgeons will be largely in demand for the remedying of the defective teeth of those who wish to join the Army at the present time, and there is no doubt that the services of the special dental departments of the hospitals will be largely taken up with this new work for the next few months. Consequently there will not be so many experts available for the needs of the general public for the time being. Moreover, as everyone knows, Mr. Newland-Pedley, consulting dental surgeon to Guy's, has gone abroad for the special purpose of forming a centre for attending to the dental requirements of French and British soldiers. Several assistants are immediately following him, and the work of this group will not only be concerned with the ordinary procedures of dentistry, but will include the care of fractured jaws. Injury to the jaws is, of course, not an uncommon result of rifle and shell fire, and proper attention to fractures of this kind makes all the difference between health and invalidism in later life, whilst improper first-aid may very likely result in fatal sepsis. In private life the services of a large number of dentists are likely to be called upon for the assistance of recruits, and it is understood that a good response has followed the request for practitioners for this work made by the British Dental Association soon after the outbreak of war.

### Sanitary Services.

The civil sanitary services will have an excellent representative in Dr. A. Newsholme, C.B., who has mobilised in the sanitary department of the

R.A.M.C., Territorial Branch, in which he holds the commission of lieutenant. It is understood that Dr. Newsholme will be assisted by a large number of medical men specialised in hygiene in the administration of the Territorial sanitary department.

An Unfavourable Outlook.

The best that can be hoped in regard to the deficiencies of the special departments thus occasioned is that a sufficient number of senior clinical assistants will be left to carry on their work. But even these usually hard-working officers lack the maturer experience that necessarily distinguishes physicians and surgeons actually in responsible charge of the various specialities at the hospitals. It is well known to students of administrative medicine that it is possible for men to be appointed to staff appointments too early, because without many years of routine work none can acquire the broad view essential to first-class expert work and even more necessary to the best teaching of special subjects. Thus it may happen that if the war be prolonged and the drain on hospital staffs steadily increases, the standard of work in the special branches, both clinical and educational, must inevitably fall below its present high value, and that without any blame attaching to those who are called up to take the places of their seniors at the present juncture. If it does happen that the special departments suffer in this way, the unfortunate sequence must be accepted by civilisation as one of those set-backs in the scientific world inseparable from a great European war.

